# Correlates of opium use: retrospective analysis of a survey of tribal communities in Arunachal Pradesh, India

**DOI:** 10.1186/1471-2458-13-325

**Published:** 2013-04-10

**Authors:** Himanshu K Chaturvedi, Jagadish Mahanta, Ram C Bajpai, Arvind Pandey

**Affiliations:** 1 National Institute of Medical Statistics, ICMR, Ansari Nagar, New Delhi, India; 2 Regional Medical Research Centre, N. E. Region, Dibrugarh, Assam, India

**Keywords:** Drug abuse, Epidemiology, Ethnic factors, India, Opium, Sex factors

## Abstract

**Background:**

Household survey data of Changlang district, Arunachal Pradesh, were used in the present study to assess the prevalence of opium use among different tribes, and to examine the association between sociodemographic factors and opium use.

**Methods:**

A sample of 3421 individuals (1795 men and 1626 women) aged 15 years and older was analyzed using a multivariate logistic regression model to determine factors associated with opium use. Sociodemographic information such as age, education, occupation, religion, ethnicity and marital status were included in the analysis.

**Results:**

The prevalence of opium use was significantly higher (10.6%) among men than among women (2.1%). It varied according to age, educational level, occupation, marital status and religion of the respondents. In both sexes, opium use was significantly higher among Singpho and Khamti tribes compared with other tribes. Multivariate logistic regression indicated that opium use was significantly associated with age, occupation, ethnicity, religion and marital status of the respondents of both sexes. Multivariate rate ratios (MRR) for opium use were significantly higher (4–6 times) among older age groups (≥35 years) and male respondents. In males, the MRR was also significantly higher in respondents of Buddhist and Indigenous religion, while in females, the MRR was significantly higher in Buddhists. Most of the female opium users had taken opium for more than 5 years and were introduced to it by their husbands after marriage. Use of other substances among opium users comprised mainly tobacco (76%) and alcohol (44%).

**Conclusions:**

The study reveals the sociodemographic factors, such as age, sex, ethnicity, religion and occupation, which are associated with opium use. Such information is useful for institution of intervention measures to reduce opium use.

## Background

The harmful effects of addictive substances have been widely documented [1-4]. Opium intake and opium dependence cause major damage to health, increase the risk of cancer, and can lead to injury, obesity, and a poor quality of life [3-7]. The consumption of opium results in substantial morbidity and mortality [1,3-8]. Despite these adverse effects on health, opium continues to be consumed by various means (e.g., smoking, drinking and swallowing) in many parts of the world, including in Southeast Asian countries [9-11]. Though non-medical use of all intoxicating substances is prohibited in India, opium has been reported to be used in many Indian States, such as Arunachal Pradesh, Assam and Rajasthan [3,10-13]. In Arunachal Pradesh, opium has been traditionally used medically among the tribal community for insomnia, pain, diarrhea, physical and mental stress, etc. It is also used socially on various celebratory occasions, such as births, marriages and festivals [10,12].

Arunachal Pradesh is the largest state in Northeast India, and shares long international borders with China and Myanmar. Drug trafficking across the border in this region has been a major problem because of the close proximity of Myanmar, which is notorious for bulk production and nodal distribution of opium and its derivatives [9,14-17]. Evidence of opium use in Arunachal Pradesh has been reported previously [10,12], but there is a need for an in-depth analysis of existing data to understand the differentials and determinants of opium use in the population. Therefore, the present study aimed to assess the prevalence of opium use among different tribes in Arunachal Pradesh and to examine the sociodemographic factors associated with opium use.

## Methods

### Study area

According to the 2011 Census, the population of Arunachal Pradesh was 1,382,611 with a density of 17 persons per km^2^. There are a large number of tribes in the State, and each lives in a specific geographical location. They mostly remain confined within their own communities, speak their own native language, and follow a distinct culture and tradition [18,19].

A survey on substance use was conducted in the Changlang district of the State (1998–2000) [12]. The population of the district was about 125,422, with 36% tribal population in the 2001 Census (see Additional file 1). The major tribes of the district are Tangsa, Singpho, Khamti and Tutsa. Of these, the Tangsa tribe has the largest population and accounts for 45% of the total tribal population in the district, followed by the Singpho tribe (7.5%), and the Tutsa and Khamti tribes (8.7%). The Tangsa mostly live together in clusters particularly at high altitude and traditionally worship supernatural powers and nature deities, but have gradually started following other religions such as Hinduism, Buddhism and Christianity. The Singpho tribe mostly lives in foothill areas close to rivers in the region, follow Buddhism, and traditionally practice meditation [19]. Khamti is the only tribe with their own script and knowledge of the medicinal value of plants. Culturally, they are close to Singpho and follow Buddhism. Another tribe of the district is the Tutsa who live in some isolated regions toward the high hills and follow different cultural beliefs and practices. Each of these tribes lives in its own group within a geographical boundary and does not intermarry. They are distinguished by their racial appearance and colorful traditional style of dress.

A list of the census villages of Changlang district was used as the sampling frame for the substance use survey. A total of 65 villages were selected randomly and survey households in each village were selected using the method of systematic sampling. The required sample size and detailed survey plan was described elsewhere [12]. Primary Health Center staff working in the community were trained to collect the information through a structured questionnaire. The head or most senior person of the household was interviewed to collect general household information. Individual information on opium use was recorded by interviewing each individual aged 15 years or older in the household. An individual who was currently consuming opium in any form and was a regular user for at least one month previously, was recorded as an opium user. Household members were interviewed separately to maintain privacy and accuracy of information regarding opium and other substance use, age of initiation and their relationship with other opium users in the family, if any. General information on ethnicity, religion, education, demographic and socioeconomic factors was also collected. During the survey, opium users underwent counseling by the interviewer individually and were given appropriate advice [12].

Sample data of 3421 individuals aged ≥ 15 years were extracted from the substance use survey (1998–2000) in Changlang district. The original data contained the records of 5135 individual respondents collected from 1092 households in 65 villages. Of these 65 selected villages, Tangsa were concentrated in 34 villages, Singpho in 11 villages, Khamti in 5 villages and Tutsas in 3 villages. The sample proportion of these tribes, e.g., Tangsa (13%), Singpho (12%), Tutsa and Khamti (10%) were similar to their proportion of the population in the district (see Additional file 1). Other tribes (Lisu, etc.) were also recorded in some samples of the villages, but they were not included in the analysis as none used opium.

### Data description and analysis

Sociodemographic information, such as age, education, and occupation of respondents, was categorized to assess any association with the prevalence of opium use. Age was categorized into three groups as 15–34, 35–54 and ≥ 55 years. Occupation was categorized as unemployed, self-employed (businessman, farmer, professional) and employed (job with regular income, pensioner). The religion of the tribes was recorded as Hindu, Christian, Buddhist and Indigenous. The education of the respondents was categorized as illiterate, primary, middle, and secondary or above.

The analysis included 1795 male and 1626 female respondents with mean age ± standard deviation (SD) of 34.3 ± 16.7 years and 33.3 ± 15.3 years, respectively. Sociodemographic differences in the prevalence of opium use in the different tribes were analyzed and compared using the Chi-square test. Multivariate logistic regression analysis [20] was applied to identify the factors associated with opium use. Odds ratios were estimated using the multivariate logistic regression model and named as multivariate rate ratios (MRR). The variables included in the multivariate model to study the factors associated with opium use were age, education, occupation, ethnic group, marital status, religion and household size of respondents. As opium use among female respondents was low, some of the sub-groups were merged or dropped to improve the estimates of odds ratios. The data were analysed using *IBM SPSS statistics for windows version 19.0* (Armonk, NY, USA).

### Ethical review

The survey was conducted by the Regional Medical Research Centre after obtaining approval from the Scientific Advisory Committee constituted by the Indian Council of Medical Research and ethical requirements were followed. The consent of the village head was obtained before conducting the survey, as well as individual consent from the household before interview [12].

## Results

The general profile of the respondents, including demographics, education, occupation, and religion is presented in Table 1. More than 50% of respondents were illiterate, 45% were self-employed (mainly farming), and the majority of unemployed respondents were women, usually working as housewives. The prevalence of opium use was recorded as 10.6% among men and 2.1% among women (*p* < 0.01). As opium use among women was low, the prevalence of opium use in each of the tribes by age, education, occupation, marital status, religion and family size is presented only for male respondents in Table 2. Opium use among men in different tribes was 23.9% in Singpho, 28.4% in Khamti, 8.6% in Tutsa and 7.8% in Tangsa. Opium use was higher in older age groups and in men with a low education level. The prevalence of opium use was high among those of Buddhist (19.2%) or Indigenous (16.9%) religions (Table 2). The sociodemographic factors associated with the prevalence of opium use were similar in all tribes.

**Table 1:** General sociodemographic characteristics of tribal respondents and age of initiation and duration of opium use

**Background characteristics**	**Male N (%)**	**Female N (%)**	**Total N (%)**
**Total number of respondents**	1795 (52.5)	1626 (47.5)	3421 (100.0)
**Mean age of respondents (years) ± SD**	34.3 ± 16.7	33.3 ± 15.3	33.8 ± 16.1
**Education**			
Illiterate	710 (39.6)	1135 (69.8)	1845 (53.9)
Primary	371 (20.7)	145 (8.9)	516 (15.1)
Middle	586 (32.6)	313 (19.3)	899 (26.3)
Secondary or above	128 (7.1)	33 (2.0)	161 (4.7)
**Occupation**			
Unemployed	476 (26.5)	1214 (74.7)	1690 (49.4)
Self employed	1149 (64.0)	382 (23.5)	1531 (44.8)
Employed	170 (9.5)	30 (1.8)	200 (5.8)
**Marital status**			
Unmarried	762 (42.5)	489 (30.1)	1251 (36.6)
Married	966 (53.8)	974 (59.9)	1940 (56.7)
Widower/Widow	67 (3.7)	163 (10.0)	230 (6.7)
**Religion**			
Buddhist	438 (24.4)	393 (24.2)	831 (24.3)
Christian	386 (21.5)	356 (21.9)	742 (21.7)
Hindu	746 (41.6)	691 (42.5)	1437 (42.0)
Indigenous	225 (12.5)	186 (11.4)	411 (12.0)
**Ethnic group**			
Khamti	74 (4.1)	58 (3.5)	132 (3.9)
Singhpho	214 (11.9)	190 (11.7)	404 (11.8)
Tangsa	1402 (78.1)	1313 (80.8)	2715 (79.4)
Tutsa	105 (5.8)	65 (4.0)	170 (4.9)
**Opium user**	191 (10.6)	34 (2.1)	225 (6.6)
**Other substance use**	Among opium user		
Tobacco (in any form)	150 (78.5)	21 (61.8)	171 (76.0)
Alcohol	93 (48.7)	6 (17.6)	99 (44.0)
		Mean ± SD (in Years)	
**Age at initiation of opium use**	23.2 ± 9.3	27.0 ± 9.9	23.7 ± 9.3
**Duration of taking opium**	3.5 ± 0.9	3.4 ± 0.9	3.4 ± 0.9

*N* Number of respondents; *SD *standard deviation.

**Table 2:** Sociodemographic differences in the prevalence and pattern of opium use among males in different tribes of Arunachal Pradesh

**Variables**	**Khamti**		**Singhpho**		**Tangsa**		**Tutsa**		**Total**	
	**N**	**Opium user (%)**	**N**	**Opium user (%)**	**N**	**Opium user (%)**	**N**	**Opium user (%)**	**N**	**Opium user (%)**
**Age (years)**										
15–34	40	10	123	12.2	823	3.9	68	-	1054	4.8
35–54	24	45.8a	58	41.4a	366	10.9	23	17.4	471	16.8
≥55	10	60.0a	33	36.4a	213	17.8	14	35.7	270	22.6
**χ**^**2 **^**(d.f. =2)**		***p*** **< 0.001**		***p*** **< 0.001**		***p*** **< 0.001**		**-**		***p*** **< 0.001**
**Education**										
Illiterate	14	50.0a	56	39.3a	595	11.4	45	15.6	710	14.6
Primary	13	30.8ab	57	38.6a	277	7.6	24	-	371	12.7
Middle	43	20.9b	85	8.2	425	4.0a	33	6.1	586	6.0a
Secondary or above	4	25.0ab	16	-	105	3.8a	3	-	128	3.9a
**χ **^**2 **^**(d.f. =3)**		***p*** **< 0.05**		***p*** **< 0.001**		***p*** **< 0.001**		**-**		***p*** **< 0.001**
**Occupation**										
Unemployed	21	-	58	1.7	364	0.6	33	-	476	0.6
Employed	8	-	15	-	134	7.5a	13	15.4	170	7.1
Self employed	45	46.7	141	35.5	904	10.8a	59	11.9	1149	15.3
**χ **^**2 **^**(d.f. =2)**		**-**		**-**		***p*** **< 0.001**		**-**		***p*** **< 0.001**
**Marital status**										
Unmarried	30	3.3	88	4.6	581	2.1	63	-	762	2.2
Married	43	46.5	115	36.5a	740	11.4	38	18.4	966	15.8
Widower	1	-	11	45.5a	51	27.5	4	50	67	31.3
**χ **^**2 **^**(d.f. =2)**		**-**		***p*** **< 0.001**		***p*** **< 0.001**		**-**		***p*** **< 0.001**
**Religion**										
Buddhist	74	28.4	214	23.9	151	8.0a	-	-	438	19.2a
Christian	-	-	-	-	386	2.9	-	-	386	2.9
Hindu	-	-	-	-	692	7.4a	54	13	746	7.8
Indigenous	-	-	-	-	173	20.8	51	3.9	225	16.9a
**χ **^**2 **^**(d.f. =3)**		**-**		**-**		***p*** **< 0.001**		**-**		***p*** **< 0.001**
**Household size**										
2–3 (Small)	8	25.0a	27	48.2	266	9.0a	11	18.2a	312	13.1a
4–6 (Medium)	34	23.5a	108	25.9	627	10.1a	40	10.0ab	809	12.7a
≥ 7 (Large)	32	34.4a	79	12.7	509	4.5	54	5.6b	674	7.0
**χ **^**2 **^**(d.f. =2)**		*p >* 0.05		***p*** **< 0.001**		***p*** **< 0.05**		***p*** **< 0.05**		***p*** **< 0.001**
**Total**	**74**	**28.4**	**214**	**23.8**	**1402**	**7.8**	**105**	**8.6**	**1795**	**10.6**

a,b: values marked by similar letter (a or b not in superscript) shows no significance difference; **χ**^**2 **^– Chi-square; d.f.- degree of freedom.

Multivariate logistic regression was used to examine the association of prevalence of opium use among male and female respondents of all tribes with age, occupation, marital status, religion, ethnicity and family size (Table 3). Among men, the MRR for opium use was significantly higher in older age groups (3.9 for 35–54 years and 5.7 for ≥ 55 years) with reference to the 15–34-year age group. MRR was also significantly higher for employed (4.1) and self-employed (8.7) male opium users than unemployed males, indicating the greater likelihood of opium use among those who had a source of income. Opium use was also significantly higher in married men (3.2) and widowers (5.4). Compared with Christians, MRR was significantly higher in Indigenous (8.3), Hindu (3.3) and Buddhist (2.8) religions. The MRR of opium use was significantly higher in Singpho and Khamti tribes (4.2 and 6.6, respectively) compared with the Tangsa tribe. The effect of education and household size on opium use was found weak in men and also in women. Among women, opium use was significantly higher in Singpho (13.2%) and Khamti (3.4%) tribes compared with the others. It was also higher among Buddhists (6.9%), with significantly higher MRR (10.1) compared with Hindus. However, none of the Christian women was recorded as an opium user. Opium use was higher among illiterate (2.6%) than literate women (0.8%), but the MRR was not significant. Opium use among older women (≥ 55 years) was also high (5.6%) with reference to the youngest age group (15–34 years) and the MRR was significant (4.3).

**Table 3:** Multivariate logistic regression analysis and prevalence of male and female opium users across various sociodemographic variables with multivariate rate ratios and 95% confidence interval

**Variables**	**Male**				**Female**			
	**N**	**Opium user (%)**	**MRR**	**95% CI**	**N**	**Opium user (%)**	**MRR**	**95% CI**
**Age (years)**								
15–34	1054	4.8	1 (Ref.)		956	0.6	1 (Ref.)	
35–54	471	16.8	**3.9f**	**2.7 *– *5.7**	457	3.5	2.7	0.9*–*8.2
≥ 55	270	22.6	**5.7f**	**3.9 *– *8.6**	213	5.6	**4.3e**	**1.1 *– *16.8**
**Education**								
Illiterate	710	14.6	1.2	0.4*–*3.4	1135	2.6	0.9	0.3*–*3.4
Literate	-	-	-	-	491	0.8	1 (Ref.)	
Primary	371	12.7	1.5	0.5*–*4.2	-	-	-	-
Middle	586	6	1.1	0.4*–*2.9	-	-	-	-
Secondary or above	128	3.9	1 (Ref.)		-	-	-	-
**Occupation**								
Unemployed	476	0.6	1 (Ref.)		252	-	-	-
Self employed	1149	15.3	**8.7f**	**2.5 *– *30.3**	**-**	**-**	**-**	**-**
Employed	170	7.1	**4.1e**	**1.1 *– *15.9**	412	1.9	1 (Ref.)	
Housewife	-	-	**-**	**-**	962	2.7	1.4	0.6-3.1
**Ethnic group**								
Tangsa	1402	7.8	1 (Ref.)		1313	0.5	1 (Ref.)	
Tutsa	105	8.6	0.9	0.4*–*1.9	65	-	-	
Singpho	214	23.8	**4.2f**	**2.1 *– *8.4**	190	13.2	**28.3f**	**12.0 *– *66.4**
Khamti	74	28.4	**6.6f**	**2.9 *– *15.2**	58	3.4	**6.7f**	**1.4 *– *32.8**
**Marital status**								
Unmarried	762	2.2	1 (Ref.)		489	-	-	-
Married	966	15.8	**3.2f**	**1.7 *– *6.2**	974	2.7	1 (Ref.)	
Widower/widow	67	31.3	**5.4f**	**2.2 *– *13.5**	163	4.9	**5.2f**	**3.0 *– *9.1**
**Religion**								
Christian	386	2.9	1 (Ref.)		356	**-**	-	
Buddhist	438	19.2	**2.8e**	**1.2 *– *6.5**	393	6.9	**10.1f**	**3.9 *– *26.5**
Hindu	746	7.8	**3.3f**	**1.7 *– *6.4**	691	0.7	1 (Ref.)	
Indigenous	225	16.9	**8.3f**	**4.3 *– *8.1**	186	1.1	1.5	0.3*–*7.8
**Household size**								
2–3 (Small)	312	13.1	1.3	0.8*–*2.1	326	1.2	0.7	0.2*–*2.3
4–6 (Medium)	809	12.7	**1.5e**	**1.1 *– *2.3**	727	2.8	1.6	0.7*–*3.4
≥ 7 (Large)	674	7	1 (Ref.)		573	1.8	1 (Ref.)	
**Total**	**1795**	**10.6**			**1626**	**2.1**		

*MRR *Multivariate rate ratios; *CI *Confidence interval; e*: p <* 0.05; f*: p <* 0.01; *Ref*. Reference category.

Overall, the mean age ± SD of initiation of opium use was 23.7 ± 9.3 years, and was significantly different between male (23.2 ± 9.3 years) and female (27.0 ± 9.9 years) opium users (*p* < 0.01). The mean duration of opium use was 3.4 ± 0.9 years, and was almost the same for men (3.5 ± 0.9 years) and women (3.4 ± 0.9 years). The correlation between age of opium users and duration of taking opium regularly was highly significant (*r* = 0.485; *p* < 0.001). The prevalence of use of other substances among opium users was recorded as 76% for tobacco in any form (chewing or smoking or both) and 44% for alcohol (Table 1).

## Discussion

The present analysis showed a strong association between opium use and age, occupation, marital status, religion and ethnicity in both sexes. There was a weak association in the case of education and household size. An association of sociodemographic factors with opium and other drug use has previously been reported [9,12,21-28]. The prevalence of opium use among men was significantly higher than among women, as previously reported by others [12,13,26,29]. However, the prevalence was found to increase with age in both sexes. The prevalence of opium use among older age groups was significantly higher compared with younger age groups. Though opium use among younger subjects was recorded as low, most of the opium users started it at a young age as reflected by a mean age of initiation of 23.2 years in men and 27 years in women. Such findings were widely reported elsewhere [12,13,25,30,31]. It is possible that there was under reporting of opium use in young age respondents because of concerns of privacy [12,26].

Though opium use was recorded in all the tribes, it was significantly higher among Khamti and Singpho tribes of this region, who were mostly Buddhist. Among the majority Tangsa tribal community, opium use was particularly high among those of Indigenous religion. In contrast, there was a low prevalence of opium use among Christians, indicating the influence of religion and the traditional culture of different tribes on their habit of opium use as discussed by others [12,25,32]. A decreasing prevalence of opium use with increasing level of education was observed, but the multivariate analysis showed that the effect of education was not significant. The lack of a major influence of education on opium use was possibly related to the strong hold of traditional beliefs and practices among tribal people. Occupation was also found to be an important correlate of opium use. Analysis revealed high opium use among employed and self-employed respondents, who had significantly higher MRRs compared with unemployed respondents. Employment status may be a proxy indicator of respondent income and their ability to afford opium. As the cost of opium is high, its affordability and the income of individual users were also important factors related to its use. An association between substance use and occupation and income has also been reported by others [10-12,25,33-35].

As mentioned earlier, only a small percentage of women (2.1%) had been using opium. As they had limited social interaction, familial influence was likely the main cause of opium use among women as in previous studies [9,12,22]. In contrast, previous studies showed that male opium users were mainly introduced or influenced by their friends at the initial stage of taking opium [10,11,23,26]. At a later stage, they continued the habit of taking opium in their own house with the acceptance of the family. As mentioned by the family during the survey, it was accepted that they control excessive opium use and prevent social harm. However, opium use had an adverse effect on their family members, especially women or female partner. Women were initially involved in helping the men prepare the opium for smoking. Thereafter, they were encouraged to use it as a medicine for body pain and stress [10,12]. Gradually, they were persuaded to join their husband in taking opium. Such an influence of the familial environment on substance use among the family of opioid and other illicit drug users was also reported by others [12,25,30].

Though the study provides useful information, it has some limitations. It was an analysis of secondary data and some categories of explanatory variables did not have an adequate number of observations to estimate the odds ratios. For instance, because of the small numbers of women in some categories, it was not possible to estimate odd ratios for the association between opium use and some independent variables.

## Conclusions

In conclusion, the study revealed a high prevalence of opium use among tribal communities in Arunachal Pradesh, and the important correlates were age, sex, occupation and religion. It suggests the need for a large population-based survey on addictive substance use, and formulation of effective interventions to curb the practice. Tribal youths can be motivated to avoid drug use by educating them about its harmful effects, and through counseling by their youth leaders and religious heads. Overall, the study provides valuable information for future community-based studies on opium use in Arunachal Pradesh.

## Competing interests

The authors declare that they have no competing interests.

## Authors’ contributions

HKC contributed to the conception, analysis, and interpretation of the data and to the writing of the paper. JM contributed to study conception and provided the data collected on substance use. RCB contributed to data analysis and preparation of the draft paper. AP contributed to interpretation of the data and to writing of the paper. All authors read and approved the final manuscript.

## Pre-publication history

The pre-publication history for this paper can be accessed here:

http://www.biomedcentral.com/1471-2458/13/325/prepub

## Supplementary Material

Additional file 1 The Census data of Changlang district of Arunachal Pradesh, India. Click here for file

## References

[B1] Galea S Nandi A Vlahov D The social epidemiology of substance use Epidemiol Review 2004 26 36 52 10.1093/epirev/mxh007 15234946

[B2] Stevenson JS Alcohol use, misuse, abuse, and dependence in later adulthood Annu Rev Nurs Res 2005 23 245 280 16350768

[B3] Pawan M Choudhary R Mathur R Choudhary MR Kamla M Study on harmful effects of opium on liver and lungs in chronic opium addicts of western Rajasthan J Bangladesh Soc Physiol 2011 6 122 126

[B4] Kato I Zeleniuch-Jacquotte A Toniolo PG Akhmedkhanov A Koenig K Shore RE Psychotropic medication use and risk of hormone-related cancers: the New York University Women’s Health Study J Public Health Med 2000 22 155 160 10.1093/pubmed/22.2.155 10912553

[B5] Challier B Chau N Predine R Choquet M Legras B Associations of family environment and individual factors with tobacco, alcohol, and illicit drug use in adolescents Eur J Epidemiol 2000 16 33 42 10.1023/A:1007644331197 10780340

[B6] Torrens M Domingo-Salvany A Alonso J Castillo C Luís S Methadone and quality of life Lancet 1999 353 1101 10199379 10.1016/S0140-6736(05)76462-X

[B7] Virk S Schwartz TL Jindal S Nihalani N Jones N Psychiatric medication induced obesity: an aetiologic review Obes Rev 2004 5 167 170 10.1111/j.1467-789X.2004.00141.x 15245385

[B8] Baumann M Spitz E Guillemin F Ravaud JF Choquet M Associations of social and material deprivation with tobacco, alcohol, and psychotropic drug use, and gender: a population-based study Int J Health Geogr 2007 6 50 10.1186/1476-072X-6-50 17996098 PMC2211297

[B9] Suwanwela C Poshyachinda V Drug abuse in Asia Bull Narc 1986 38 41 53 3535959

[B10] Mahanta J Chaturvedi HK Phukan RK Opium addiction in Assam: a trend analysis Indian J Psychiatry 1997 39 143 146 21584061 PMC2967099

[B11] Mohan D Sundaram KR Sharma HK A study of drug abuse in rural areas of Punjab (India) Drug Alcohol Depend 1986 17 57 66 10.1016/0376-8716(86)90036-0 3487439

[B12] Chaturvedi HK Mahanta J Sociocultural diversity and substance use pattern in Arunachal Pradesh, India Drug Alcohol Depend 2004 74 97 104 10.1016/j.drugalcdep.2003.12.003 15072813

[B13] Lakshminarayana J Singh MB Opium addiction among rural population in desert districts of Western Rajasthan: some observations from the study J Hum Ecol 2009 25 1 4

[B14] Wairagkar NS Das J Kumar S Mahanta J Satyanarayana K Codeine containing cough syrup addiction in Assam and Nagaland Indian J Psychiatry 1994 36 129 132 21743687 PMC2972479

[B15] Kumar S Wairagkar NS Mahanta J Satyanarayan K Chetia M Profile of heroin addicts in Nagaland, India Southeast Asian J Trop Med Public Health 1996 27 768 771 9253882

[B16] Khant U Measures to prevent and reduce drug abuse among young people in Burma Bull Narc 1985 37 81 89 2934106

[B17] Poshyachinda V Drug injecting and HIV infection among the population of drug abusers in Asia Bull Narc 1993 45 77 90 8305908

[B18] Panchani CS Arunachal Pradesh: Religion 1989 Culture and Society: Delhi, Konark Publication

[B19] Nair PT Tribes of Arunachal Pradesh 1985 Michigan: Spectrum publication

[B20] Hosmer DW Lemeshow S Applied logistic regression 2000 New York: John Wiley & Sons

[B21] Miller PM Martin P Drinking, smoking, and illicit drug use among 15 and 16 year olds in the United Kingdom BMJ 1996 313 394 397 10.1136/bmj.313.7054.394 8761226 PMC2351821

[B22] Charles M Masihi EJ Siddiqui HY Jogarao SV D’Lima H Culture, drug abuse and some reflection on the family Bull Narc 1994 46 67 86 7833904

[B23] Seck B Choquet M Sarr L Gueye M Attitude and behaviour of the youth of Senegal towards drugs Dakar Med 1994 39 17 22 7493515

[B24] Neumark YD Rahav G Jaffe H Socio-economic status and binge drinking in Israel Drug Alcohol Depend 2003 69 15 21 10.1016/S0376-8716(02)00248-X 12536062

[B25] Westermeyer J Sex differences in drug and alcohol use among ethnic groups in Laos, 1965–1975 Am J Drug Alcohol Abuse 1988 14 443 461 10.3109/00952998809001563 3232678

[B26] Chaturvedi HK Phukan RK Mahanta J The association of selected sociodemographic factors and differences in pattern of substance use: a pilot study in selected areas of Northeast India Subst Use Misuse 2003 38 1305 1322 10.1081/JA-120018488 12908813

[B27] Gureje O Degenhardt L Olley B Uwakwe R Udofia O A descriptive epidemiology of substance use and substance use disorders in Nigeria during the early 21^st^ century Drug Alcohol Depend 2007 91 1 9 10.1016/j.drugalcdep.2007.04.010 17570618

[B28] Tominaga M Kawakami N Ono Y Nakane Y Tachimori YH Prevalence and correlates of illicit and non-medical use of psychotropic drugs in Japan: findings from the World Mental Health Japan Survey 2002–2004 Soc Psychiatry Epidemiol 2009 44 777 783 10.1007/s00127-009-0499-1 19190833

[B29] Back SE Payne RL Simpson AN Brady KT Gender and prescription opioids: findings from the National Survey on Drug Use and Health Addict Behav 2010 35 1001 1007 10.1016/j.addbeh.2010.06.018 20598809 PMC2919630

[B30] Ahmadi J Arabi H Mansouri Y Prevalence of substance use among offspring of opioid addicts Addict Behav 2003 28 591 595 10.1016/S0306-4603(01)00260-X 12628630

[B31] Zarnaghash M Goodarzi MA Study of the effect of family patterns style on opium abusers, cigarette abusers and normal group Procedia Social and Behavioral Sciences 2010 5 78 81

[B32] Barrett ME Correlates of illicit drug use in Karen villages in Northern Thailand Subst Use Misuse 2003 38 1615 1649 10.1081/JA-120024233 14582572

[B33] Ghulam R Rahman I Naqvi S Gupta SR An epidemiological study of drug abuse in urban population of Madhya Pradesh Indian J Psychiatry 1996 38 160 165 21584124 PMC2970835

[B34] Lapeyre-Mestre M Sulem P Niezborala M Taking drugs in the working environment: a study in a sample of 2106 workers in the Toulouse metropolitan area Therapie 2004 59 615 623 10.2515/therapie:2004107 15789825

[B35] Tolone WL Dermott D Some correlates of drug use among high school youth in mid western rural community Int J Addict 1975 10 761 777 1176230 10.3109/10826087509027336

