# Supplementary material for: Correlates of opium use: retrospective analysis of a survey of tribal communities in Arunachal Pradesh, India
Source: BMC Public Health. 2013 Apr 10;13:325. doi: 10.1186/1471-2458-13-325 (PMC3626656; doi:10.1186/1471-2458-13-325)
Supplement: Additional file 1 — The Census data of Changlang district of Arunachal Pradesh, India. [file 1471-2458-13-325-S1.pdf]

# Basic Data Sheet

## District Changlang (12), Arunachal Pradesh (12)

*( Source: Census of India 2001)*

**Population:**

| | |
|---|---|
| Persons | 125,422 |
| Males | 65,821 |
| Females | 59,601 |
| Growth (1991 - 2001) | 30.84 |
| Rural | 113,034 |
| Urban | 12,388 |
| Scheduled Caste population | 372 |
| Percentage to total population | 0.30 |

| | |
|---|---|
| Number of households | 24,012 |
| Household size (per household) | 5 |
| Sex ratio (females per 1000 males) | 906 |
| Sex ratio (0-6 years) | 954 |
| Scheduled Tribe population | 45,351 |
| Percentage to total population | 36.16 |

**Literacy and Educational level**

| | |
|---|---|
| *Literates* | |
| Persons | 51,291 |
| Males | 32,789 |
| Females | 18,502 |
| *Literacy rate* | |
| Persons | 51.32 |
| Males | 62.13 |
| Females | 39.23 |

| | |
|---|---|
| *Educational Level attained* | |
| Total | 51,291 |
| Without level | 1,311 |
| Below primary | 18,470 |
| Primary | 13,700 |
| Middle | 8,179 |
| Matric/Higher Secondary/Diploma | 7,679 |
| Graduate and above | 1,952 |

**Workers**

| | |
|---|---|
| Total workers | 60,045 |
| Main workers | 49,176 |
| Marginal workers | 10,869 |
| Non-workers | 65,377 |

**Age groups**

| | |
|---|---|
| 0 - 4 years | 17,342 |
| 5 - 14 years | 34,197 |
| 15 - 59 years | 67,584 |
| 60 years and above (Incl. A.N.S.) | 6,299 |

**Scheduled Castes (Largest three)**

| | |
|---|---|
| 1.Brittial-Bania etc. | 129 |
| 2.Namasudra | 56 |
| 3.Dhupi etc. | 42 |

**Scheduled Tribes (Largest three)**

| | |
|---|---|
| 1.Tangsa | 20,431 |
| 2.Any Naga Tribes etc. | 3,940 |
| 3.Singpho | 3,412 |

**Religions (Largest three)**

| | |
|---|---|
| 1.Hindus | 50,183 |
| 2.Buddhists | 42,744 |
| 3.Christians | 21,931 |

**Important Towns (Largest three)**

| | Population |
|---|---|
| 1.Changlang (CT) | 6,469 |
| 2.Jairampur (CT) | 5,919 |

**House Type**

| Type of house (% of households occupying) | |
|---|---|
| Permanent | 14.9 |
| Semi-permanent | 11.1 |
| Temporary | 73.9 |

**Amenities and infrastructural facilities**

**Total inhabited villages** 336

Amenities available in villages

| | No. of villages |
|---|---|
| Drinking water facilities | 336 |
| Safe Drinking water | 307 |
| Electricity (Power Supply) | 277 |
| Electricity (domestic) | 258 |
| Electricity (Agriculture) | 1 |
| Primary school | 179 |
| Middle schools | 47 |
| Secondary/Sr Secondary schools | 25 |
| College | 1 |
| Medical facility | 64 |
| Primary Health Centre | 7 |
| Primary Health Sub-Centre | 11 |
| Post, telegraph and telephone facility | 45 |
| Bus services | 64 |
| Paved approach road | 195 |
| Mud approach road | 133 |
